# Development and validation of the PipeSeq program for RNA-seq data analysis in the Chlamydomonas reinhardtii as a model

**DOI:** 10.18699/vjgb-26-34

**Published:** 2026-04

**Authors:** A.M. Nerezenko, P.A. Virolainen, S.A. Tupitsyna, E.M. Chekunova

**Affiliations:** Saint-Petersburg University, St. Petersburg, Russia; Saint-Petersburg University, St. Petersburg, Russia; Saint-Petersburg University, St. Petersburg, Russia; Saint-Petersburg University, St. Petersburg, Russia

**Keywords:** RNA sequencing, RNA-seq, RT-qPCR, pipeline, transcriptome, gene expression, GATA family transcription factors, GATA TFs, Chlamydomonas reinhardtii, cеквенирование РНК, РНК-сек, ОТ-ПЦР-РВ, пайплайн, транскриптом, экспрессия генов, факторы транскрипции семейства GATA, ФТ GATA, Chlamydomonas reinhardtii

## Abstract

RNA sequencing (RNA-seq) is a highly sensitive method for transcriptome analysis that allows simultaneous assessment of expression of thousands of genes and identification of expression patterns under various conditions. The existing variety of RNA-seq data formats, normalization methods, and approaches to statistical processing of results complicates comparison of data from different studies and reduces reproducibility of the analysis. This study presents an automated pipeline PipeSeq that combines standard steps of RNA-seq data processing: loading (SRA Toolkit), read alignment to the reference genome (HISAT2), transcript assembly (StringTie), transcript counting (FeatureCounts) and statistical analysis of differential gene expression under various experimental conditions (DESeq2). PipeSeq has a simple visual interface, supports multithreading, and generates ready-to-analyze gene expression heat maps, tables and graphs. The functionality of the pipeline is demonstrated on three sets of raw RNA-seq data from the green alga Chlamydomonas reinhardtii cells available in the NCBI SRA database. The data from these experiments were used to analyze the differential expression of C. reinhardtii genes encoding the GATA family transcription factors under different light cultivation conditions. The data obtained by in silico methods were verified by real-time reverse transcription polymerase chain reaction (RT-qPCR) for 12 GATA genes, which allowed us to hypothesize their functions and evaluate the correlation between the bulk (RNA-seq) and targeted (RT-qPCR) approaches. Our results showed that RNA-seq and RT-qPCR methods reveal similar directions of gene expression changes, but demonstrate differences in the effect size and sensitivity, which emphasizes the need for a combined use of the two approaches. Thus, the PipeSeq program is a tool for conducting a full cycle of bioinformatic analysis of RNA-seq data, additionally providing the opportunity to process RT-qPCR data and perform a comparative statistical analysis of the results obtained.

## Abstract (ru)

Секвенирование РНК (РНК-сек) – высокочувствительный метод анализа транскриптома, позволяющий одновременно оценивать экспрессию тысяч генов и выявлять паттерны экспрессии в различных условиях. Существующее разнообразие форматов данных РНК-сек, методов нормализации и подходов к статистической обработке результатов затрудняет сопоставление данных разных исследований и снижает воспроизводимость анализа. В настоящей работе представлен автоматизированный пайплайн PipeSeq, объединяющий стандартные этапы обработки данных РНК-сек – от загрузки (SRA Toolkit), выравнивания прочтений на референсный геном (HISAT2) и сборки транскриптов (StringTie) до подсчета транскриптов (FeatureCounts) и статистического анализа дифференциальной экспрессии генов в различных экспериментальных условиях (DESeq2). Программа PipeSeq имеет простой визуальный интерфейс, поддерживает многопоточность и формирует готовые для анализа тепловые карты экспрессии генов и отчеты в форме таблиц и графиков. Функциональность пайплайна продемонстрирована на трех наборах пакетов сырых данных секвенирования РНК клеток зеленой водоросли Chlamydomonas reinhardtii, доступных в открытой базе данных NCBI SRA. Результаты этих экспериментов были использованы для анализа дифференциальной экспрессии генов C. reinhardtii, кодирующих факторы транскрипции семейства GATA, в различных световых условиях культивирования. Полученные методами in silico данные верифицированы методом полимеразной цепной реакции в реальном времени с обратной транскрипцией (ОТ-ПЦР-РВ) по 12 генам GATA, что позволило выдвинуть предположения об их функциях, а также оценить степень согласованности между массовым (РНК-сек) и таргетным (ОТ-ПЦР-РВ) подходами. Результаты нашего исследования показали, что методы секвенирования РНК и ОТ-ПЦР-РВ выявляют схожие направления изменения экспрессии генов, но демонстрируют различия по оценке степени размера эффекта и чувствительности, что подчеркивает необходимость совместного применения двух подходов. Таким образом, программа PipeSeq представляет собой инструмент для проведения полного цикла биоинформатического анализа данных РНК-сек, дает возможность обрабатывать данные ОТ-ПЦР-РВ и выполнять сравнительный статистический анализ полученных результатов.

## Introduction

In recent years, RNA sequencing (RNA‑seq) has become widely adopted as a reliable approach for large‑scale quantitative analysis of gene expression across diverse biological systems (Marioni et al., 2008; Mortazavi et al., 2008; Conesa et al., 2016; Li X., Wang, 2021). Due to the “digital” nature of the data, RNA‑seq enables direct calculation of the number of reads for each transcript, providing a wide dynamic measurement range and high reproducibility of experimental results. In addition, this method makes it possible to detect previously unannotated transcripts and alternative splicing variants (Wang et al., 2009; Li X., Wang, 2021). These features establish RNA sequencing as a powerful tool for systems‑level studies of the transcriptome, enabling reliable quantification of transcript levels under various experimental conditions and identification of differential gene expression in the study object (Wang et al., 2009).

The processing, accumulation and consolidation of these data can contribute to obtaining novel insights into the functioning of living systems. To implement this approach, it is essential to address the challenge of standardizing the processing of raw RNA‑seq data (Conesa et al., 2016; Li X., Wang, 2021). Currently, outdated normalization methods are still employed for gene expression assessment. These methods are characterized by low reproducibility, fail to adequately correct for compositional biases, and hinder direct comparison of results across independent studies. Such methods include: RPKM (Reads Per Kilobase per Million mapped reads), FPKM (Fragments Per Kilobase of exon model per Million mapped fragments), and TPM (Transcripts Per Million). At present, methods based on the negative binomial distribution – DESeq2 and edgeR – are considered the standard for differential gene expression analysis. Normalized counts generated by DESeq2 have demonstrated the lowest coefficient of variation and highest reproducibility (Zhao S. et al., 2020; Zhao Y. et al., 2021; Elahimanesh, Najafi, 2024).

Comparative analysis of RNA‑seq data processing results and molecular biology data is complicated by several methodological challenges. Although RNA sequencing is recognized as a reliable method for global expression profiling, the results obtained through this technique typically represent relative changes in transcript levels across the entire genome. Historically, reverse transcription quantitative polymerase chain reaction (RT‑qPCR) has been established as the “gold standard” for validating data derived from transcriptomic studies (Derveaux et al., 2010; Coenye, 2021). However, RT‑qPCR‑based methods and RNA sequencing technology rely on different protocols, which complicates their direct quantitative comparison. Several factors can influence the obtained results, including: efficiency of reverse transcription and amplification processes, data normalization methods, sensitivity of the techniques employed.

Currently, RNA‑seq is utilized to cover the entire transcriptome of the study object and identify a set of differentially expressed candidate genes in response to a specific stimulus. In contrast, RT‑qPCR is applied for precise quantitative assessment of these changes, specifically targeting a limited pool of genes of interest (Shi, He, 2014; He et al., 2015; Coenye, 2021).

To understand the mechanisms of metabolic regulation, special attention is devoted to the study of transcription factors (TFs) as key regulators of gene activity. TFs are proteins capable of binding to specific DNA sequences in promoter regions, thereby enhancing or repressing the transcription of target genes. These regulators coordinate gene activity in response to various environmental changes and signaling influences, participating in global processes such as growth, development, and adaptation to stress factors (Riechmann et al., 2000).

In photosynthetic organisms, genes encoding GATA family transcription factors are of particular interest. GATA TFs are proteins that carry a conserved zinc finger domain of type IV (general formula: CX2CX18–20CX2C). This domain mediates binding to the consensus sequence (A/T)GATA(A/G) in the promoters of target genes (Reyes et al., 2004). Plant GATA factors are involved in the regulation of photomorphogenesis, nitrogen and carbon metabolism, and hormonal control (Manfield et al., 2007; Naito et al., 2007; Luo et al., 2010; Schwechheimer et al., 2022; Schröder et al., 2023; Ren et al., 2025). In recent years, there has been a significant increase in scientific interest in studying GATA TFs in bryophytes and algae, as their biological functions and evolutionary roles remain insufficiently characterized (Schwechheimer et al., 2022; Virolainen, Chekunova, 2024).

The aim of the present study is to develop an integrated solution for standardizing the processing of raw RNA sequencing data and RT‑qPCR results.

To achieve this goal, the following objectives must be addressed.

1. Developing an automated pipeline for conducting a full cycle of RNA‑seq data analysis. 2. Testing the pipeline on raw RNA‑seq data publicly available in the NCBI SRA database, using as a model the genes encoding GATA family TFs in a model object of photosynthesis genetics, the green alga Chlamydomonas reinhardtii, in response to changes in light growing conditions. 3. Conducting an analysis of GATA gene expression in C. reinhardtii using the RT‑qPCR method in response to changes in light conditions during growth. 4. Performing a comparative statistical analysis of the obtained RNA‑seq and RT‑qPCR data.

## Materials and methods

**Quantitative analysis of gene expression using the PipeSeq program. **To analyze the expression profile of 12 genes encoding GATA TFs in C. reinhardtii under various cultivation conditions by in silico methods, the following publicly available raw RNA‑seq data packages were selected from the NCBI SRA database (Wheeler et al., 2005): SRX8380269, SRX8380270, SRX8380271 (acclimation to high light, 600 μmol/m2/s, 1 h), SRX7413406, SRX7413407, SRX7413412, SRX7413413, SRX7413414, SRX7413415 (acclimation to light, 30 min), SRX5120530, SRX5120531, SRX5120532, SRX5120533, SRX5120534, SRX5120535 (acclimation to darkness).

The three selected datasets (PRJNA634446, PRJNA596622, PRJNA509798) include both experimental (altered growth conditions) and control (standard growth conditions) RNA‑seq data for the wild‑type strain CC-124 (wt, mt-) of C. reinhardtii performed in triplicate biological and technical replicates. For read alignment, the C. reinhardtii genome assembly (v5.6) (Merchant et al., 2007) and its annotation in GTF format were downloaded from the Phytozome portal (Goodstein et al., 2012).

Data upload and processing were carried out using Pipe‑ Seq, an automated software package consisting of 17 scripts and more than 2,000 lines of code. The tool is designed for system analysis of transcriptomic data obtained via RNA‑seq, processing of RT‑qPCR data using the ΔΔCt method (Livak, Schmittgen, 2001; Schmittgen, Livak, 2008), and comparative analysis of results (including calculation of Pearson, Spearman, and Kendall correlation coefficients). The program was developed in Python (version 3.9) using Pandas (McKinney, 2011), Matplotlib (Hunter, 2007), PyQt6 (https://www.river bankcomputing.com/software/pyqt/), PyDESeq2 (Muzellec et al., 2023) libraries, and bioinformatics tools SRA Toolkit, FastQC (https://www.bioinformatics.babraham.ac.uk/projects/ fastqc/), MultiQC (Ewels et al., 2016), Cutadapt (Martin, 2011), HISAT2 (Kim et al., 2019), SAMtools (Li H. et al., 2009), FeatureCounts (Liao et al., 2014), StringTie (Pertea et al., 2015), DESeq2 (Love et al., 2014).

**Strains and cultivation conditions. **The wild-type strain CC-124 (wt, mt-) of C. reinhardtii from the Peterhof Genetic Collection at St. Petersburg State University (Kvitko et al., 1983) was grown in Petri dishes on agarized TAP medium (1.5 %) (Harris, 1989), supplemented with arginine (50 mg per liter of medium) and yeast autolysate (4 g per liter of medium) at a temperature of 20–25 °C with a day (14 h, illumination 90 μmol/m2/s) / night (10 h) cycle and subculturing every three days. Culture samples were grown and collected under standard illumination conditions (90 μmol/m2/s) and darkness (control conditions), under conditions of increased illumination (high light, 215 μmol/m2/s) for 30 min and 2 h (transfer of cultures from standard conditions), under conditions of standard illumination for 30 min and 2 h (transfer of cultures from darkness), under darkness for 30 min and 2 h (transfer of cultures from standard conditions) (experimental conditions).

**Culture fixation and RNA isolation.** Fixation of C. reinhardtii cell cultures grown under control and experimental conditions and total RNA isolation was performed using the “ExtractRNA” reagent (Evrogen, Russia) in strict accordance with the manufacturer’s protocol. RNA preparations were treated with DNase I (Thermo Fisher Scientific, USA) to remove genomic DNA contamination, and subsequently ethanol‑precipitated. The concentration of total RNA was measured using an Eppendorf BioPhotometer plus spectrophotometer (Eppendorf, Germany).

**Primer design.** Gene‑specific primers were designed using the IDT PrimerQuest Tool (https://www.idtdna.com/pages/ tools/primerquest) and NCBI Primer-BLAST (https://www. ncbi.nlm.nih.gov/tools/primer-blast/). Primer design criteria included positioning of at least one primer at an exon–exon junction, or ensuring that an intron sequence was located between primer binding sites. The constitutively expressed genes RPL19 (ribosomal protein L19) and RPL32 (ribosomal protein L32) of C. reinhardtii were used as reference genes for data normalization (Liu et al., 2012). The RBCS (Ribulose bisphosphate carboxylase small subunit) gene of C. reinhardtii was used as experimental condition change control (Sanchez- Tarre, Kiparissides, 2021). Primer sequences are listed in the Table.

**Table 1.**
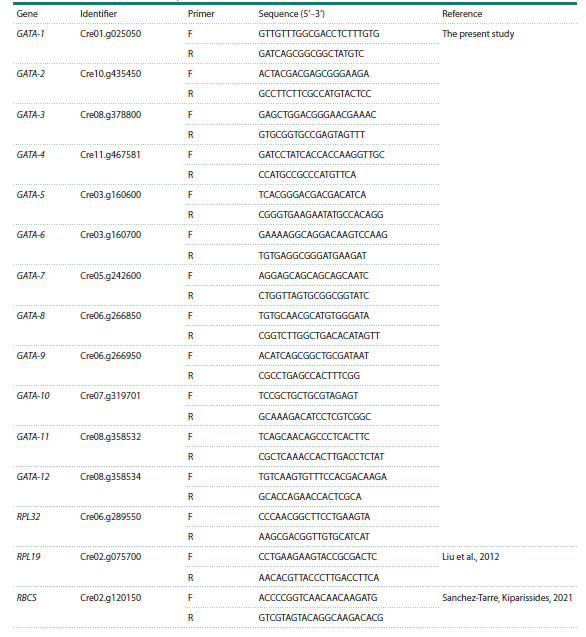
Primer sequences for quantitative analysis of GATA gene expression

Primer specificity assessment included melting curve analysis across a series of controls (no‑template control, no‑reverse‑transcription control, positive control) and visualization of PCR products via gel electrophoresis (Derveaux et al., 2010). PCR efficiency was evaluated using the software of the QuantStudio 5 real‑time PCR system (Thermo Fisher Scientific, USA).

**Quantitative analysis of gene expression by RT‑qPCR.** RT‑qPCR reactions were performed in a one‑step format using the “OneTube RT‑PCR SYBR” reagent kit (Evrogen, Russia), strictly following the manufacturer’s instructions. Amplification was carried out on a QuantStudio 5 real‑time PCR system (Thermo Fisher Scientific, USA), with fluorescence reading during the elongation and melting steps. The thermal cycling protocol was as follows: 55 °C – 15 min, 95 °C – 1 min, then 50 cycles: 95 °C – 15 s, 62 °C – 20 s, 72 °C – 20 s, melting: 55–95 °C with 0.5 °C increments. Each sample was analyzed in triplicate biological replicates (averaged) (Derveaux et al., 2010). Data processing and visualization were performed using the ΔΔCt method (Livak, Schmittgen, 2001; Schmittgen, Livak, 2008) in the developed PipeSeq program.

**Comparative analysis of RNA‑seq and RT‑qPCR results.** A comparative statistical analysis of the data obtained by different methods was carried out in the PipeSeq program

## Results

**PipeSeq pipeline development**

**Fig. 1.**
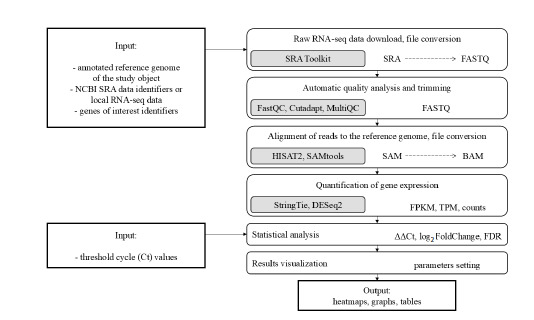
Workflow diagram of the PipeSeq program.

The aim of this work was to develop such a tool (pipeline) that would enable a full cycle of RNA‑seq data analysis via a simple visual interface, requiring minimal user involvement in system administration. The pipeline is designed to run in a local Windows environment using the Windows Subsystem for Linux (WSL) to execute Linux commands. The workflow of the PipeSeq program is presented in Figure 1.

All steps of the algorithm are fully automated and allow processing large amounts of data through optimized commands and parallel computing. The input data are FASTQ files containing raw short sequencing reads obtained after conversion from the SRA format using the SRA Toolkit included in the software package. At the initial stage, automated preprocessing of reads is performed, which includes quality control (FastQC), removal of adapter sequences, low-quality nucleotides, and short reads (Cutadapt), aggregation of summary reports (MultiQC). Next, the reads are aligned to the reference genome using the HISAT2 tool (Kim et al., 2019), with the reference genome index being automatically created if it is absent. The advantages of HISAT2 include splicing consideration, accurate determination of transcript structure, and rapid analysis of alignment quality. After alignment, files are converted from the SAM format to the BAM format, sorted, and prepared for further analysis using the SAMtools (Li H. et al., 2009).

The developed PipeSeq program integrates three normalization approaches: DESeq2 (for differential expression analysis), TPM (absolute quantification), and FPKM (an outdated metric included to ensure backward compatibility). At this stage, quantitative assessment of gene expression is performed by counting the number of reads mapping to genes and transcripts using the FeatureCounts package (Liao et al., 2014), as well as transcript assembly and calculation of FPKM and TPM values using the StringTie tool (Pertea et al., 2015). This tool analyzes read alignments, builds transcripts, and identifies exons, introns, and splice sites. The PipeSeq program provides the ability to disable the function of assembling new transcripts to minimize the likelihood of false results. These steps are performed considering various modes, such as strict annotation (the “-e” option in StringTie) and adjustable sensitivity (the “-c” parameter).

A package based on the negative binomial distribution, DESeq2 (Love et al., 2014), implemented in the PyDESeq2 library (Muzellec et al., 2023), is used for statistical analysis of differential gene expression. The PipeSeq pipeline automatically prepares input data for DESeq2, performs statistical calculations, and generates final tables with logarithmic changes in expression levels (log2FoldChange) and corresponding values of the adjusted p‑value – FDR (False Discovery Rate) using the Benjamini–Hochberg procedure. Statistical hypotheses about differential gene expression are tested based on a generalized linear model of the negative binomial distribution. After normalization for data counting and variance estimation (followed by averaging variance estimates across all genes using Bayesian shrinkage), the Wald test is applied.

The program also implements the possibility of processing RT-qPCR data using the ΔΔCt method (Livak, Schmittgen, 2001; Schmittgen, Livak, 2008) and conducting comparative statistical analysis with the three data normalization methods used in RNA‑seq (Pearson’s linear correlation coefficient, Spearman’s rank correlation coefficient, and Kendall’s rank correlation coefficient).

As output, the PipeSeq program generates heatmaps of expression reflecting changes in the expression levels of genes of interest under different experimental conditions, summary tables with log2FoldChange values, and graphs. A wide range of data display settings is available to the user. The program is available for download at the following link: https://github. com/MarvinMarss/PipeSeq.

**Processing of available RNA sequencing data of C. reinhardtii cells using the PipeSeq pipeline**

**Fig. 2.**
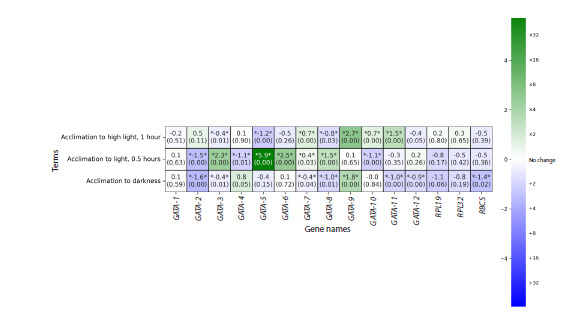
Heatmap of transcript‑level expression for 12 genes encoding GATA family transcription factors and three control genes (RPL19, RPL32, RBCS) in C. reinhardtii under various acclimation conditions according to the RNA‑seq data. Genes are reflected from left to right, experimental conditions are shown from top to bottom: acclimation to high light for 1 h (row 1), acclimation to light for 30 min (row 2), acclimation to darkness (row 3). Here and in Fig. 3: the cells are colored according to log2FoldChange values, where positive values (green scale) indicate the induction of expression, negative values (blue scale) indicate repression, and zero values (white color) signify no change in the expression level. The statistical significance of the changes was assessed by adjusted p-values with a threshold of 0.05. The control transcripts were included to verify the quality of data normalization. The images were created in the PipeSeq program.

Using the developed program, we analyzed three available RNA‑seq datasets of C. reinhardtii wild‑type strain CC-124 (wt, mt-) cells under various light conditions: acclimation to high light (600 μmol/m2/s) for 1 h, acclimation to light for 30 min, and acclimation to darkness. The generated heatmap demonstrates the complex dynamics of gene expression for GATA TFs in response to changes in light conditions (Fig. 2). Most of the obtained values of logarithmic change in gene expression levels were statistically insignificant.

Under conditions of increased illumination, a significant upregulation of the expression levels of the GATA-7, GATA-9, GATA-10, GATA-11 genes is observed, whereas transcription of the GATA-3, GATA-5, GATA-8 genes is repressed (statistically significantly). It is likely that excessively high light intensity has a negative impact on the viability of C. reinhardtii cells.

Under 30‑minute light acclimation, a metabolic rearrangement of the cells occurs, which is marked by a significant increase in transcript levels of the GATA-3, GATA-5, GATA-6, GATA-7, GATA-8 genes. In these conditions, the expression of the GATA-2, GATA-4, GATA-10 genes is repressed (statistically significantly).

In the dark, there is a statistically significant suppression of the GATA-2, GATA-3, GATA-7, GATA-8, GATA-11, GATA- 12 genes expression, along with active expression of the GATA- 9 gene.

The publicly available datasets are characterized by limited experimental conditions and poor characterization, as well as a low level of reliability of changes. We decided to use the RT‑qPCR method to obtain comprehensive data on GATA gene expression. Analysis of the RNA‑seq data allowed us to select reference genes with stable expression under varying light conditions for our own experiments. The RPL19 and RPL32 genes encoding ribosomal proteins were discovered and then confirmed by literature data (Liu et al., 2012). According to published data (Sanchez-Tarre, Kiparissides, 2021), the expression of the RBCS gene varies depending on the light spectrum and intensity. Therefore, we selected this gene as a control of changes in experimental conditions.

**Analysis of GATA gene expression in C. reinhardtii using RT‑qPCR**

**Fig. 3.**
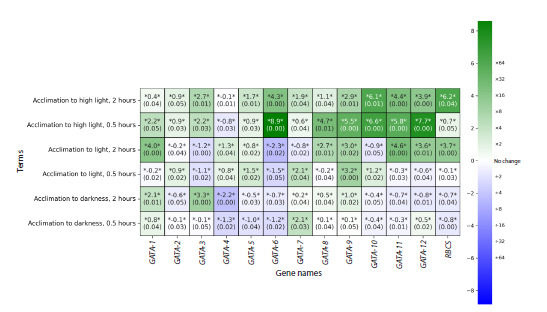
Heatmap of gene expression (at the transcriptional level) for 12 genes encoding GATA family transcription factors and the experimental condition change control gene (RBCS) in C. reinhardtii under various acclimation conditions, based on RT‑qPCR data. The data are normalized to the reference gene pair RPL19–RPL32. Genes are reflected from left to right, experimental conditions are shown from top to bottom: acclimation to high light for 2 h (row 1), for 30 min (row 2), acclimation to light for 2 h (row 3), for 30 min (row 4), acclimation to darkness for 2 h (row 5), for 30 min (row 6).

The results of GATA gene expression analysis in C. reinhardtii obtained by the RT‑qPCR method are presented in Figure 3.

The entire group of GATA genes responds to changes in light conditions rapidly and in a coordinated manner. The strongest response is observed during the first 30 min of exposure to high light intensity (215 μmol/m2/s) after transferring the culture from standard conditions (90 μmol/m2/s): nearly all genes show a multi‑fold increase in expression levels in response to the stress stimulus. Notably, significant activation of GATA-6 gene expression occurs only under these conditions. High light intensifies the metabolism of C. reinhardtii cells, driving a unified shift in the expression profile of the studied genes. After 2 h of acclimation, stabilization is observed: the expression of several genes (GATA-1, GATA-6, GATA-8, GATA-9, GATA-10, GATA-11, GATA-12) decreases significantly, while the expression level of GATA-5 and GATA-7 increases.

During the first 30 min after transferring the culture from darkness to light (90 μmol/m2/s), cell metabolism undergoes reprogramming. The transcription of the GATA-1 and GATA-3 genes is suppressed simultaneously with the activation of the GATA-2, GATA-4, GATA-5, GATA-7, GATA-9, and GATA-10 genes. By 2 h of exposure, two profiles are formed that ensure the maintenance of growth and development processes in optimal light conditions: (1) actively transcribed genes (GATA-1, GATA-4, GATA-5, GATA-8, GATA-9, GATA-11, GATA-12); (2) repressed genes (GATA-2, GATA-3, GATA-6, GATA-7, GATA-10).

In the first 30 min after culture transfer from light (90 μmol/ m2/s) to darkness, the expression of all GATA genes, with the exception of the GATA-7 gene, is suppressed. Apparently, its product is involved in the metabolic switching processes of C. reinhardtii cells during light/dark and dark/light transitions. After 2 h, cell metabolism stabilizes with the identification of three actively transcribed genes, apparently involved in ensuring adaptation to darkness – GATA-1, GATA-3, GATA-9.

The identified dynamics of GATA gene expression align with the standard model of stress response: a change in light conditions triggers a broad emergency cascade (a rapid response to the stimulus), while prolonged exposure narrows the response down to specific regulatory modules. A unique expression profile is observed under each cultivation condition, with some of the GATA genes presumably acting as “switches” between the light and dark metabolic programs.

**Comparative analysis of the obtained results**

**Fig. 4.**
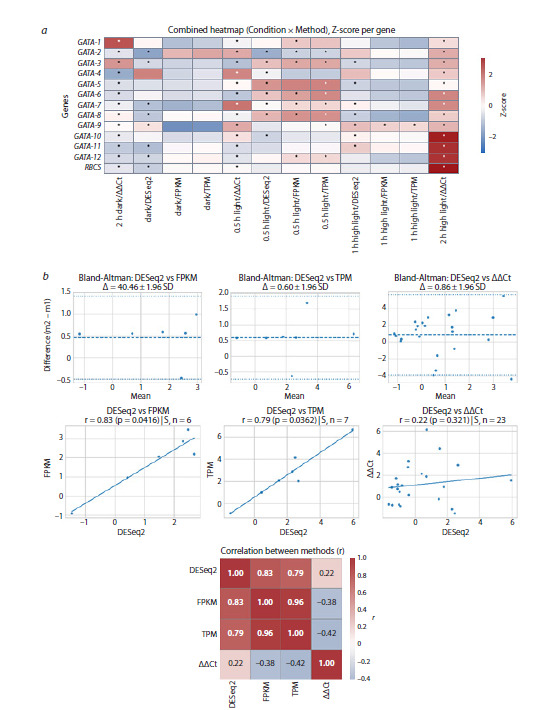
Comparative statistical analysis of three RNA‑seq normalization methods – FPKM, TPM, DESeq2 – and the ΔΔCt method for RT‑qPCR data. a, A combined heatmap of Z‑scores for log fold change in the expression (log2FoldChange) values of 12 genes encoding GATA family transcription factors and the RBCS gene in C. reinhardtii presented for each combination of experimental conditions and normalization method. Statistically significant changes (FDR < 0.05) are marked with an asterisk. b, Bland–Altman plots, correlation scatterplots, and a matrix of statistically significant (FDR < 0.05) log2FoldChange estimates across the compared normalization methods. Color reflects the Pearson correlation coefficient (r), where the red scale indicates positive correlation, and the blue scale indicates negative correlation. The images were created in the PipeSeq program.

A comparative analysis based on the results of RNA-seq data processed with three normalization methods – FPKM, TPM, DESeq2 – and RT‑qPCR data processed using the ΔΔCt method was conducted in the PipeSeq program. The constructed correlation matrix allows us to evaluate the concordance between data obtained by different methods and approaches (Fig. 4).

Cross‑platform consistency of the results is limited and context‑dependent. Despite methodological differences, reproducible agreements include induction of the GATA-7, GATA-9, GATA-10 and GATA-11 genes expression under high light conditions, repression of GATA-2, GATA-11, and GATA-12 in the dark and upregulation of GATA-9 expression in darkness. Early repression of GATA-3, GATA-5, and GATA-8 under high light observed at 1 h in RNA‑seq data appeared to be inverted in RT‑qPCR results at the 2 h time point. This pattern is consistent with a potential phase shift in the cellular response; however, confirmation requires a time‑matched dataset. In dark conditions, both datasets corroborated a common repression of photosynthesis‑related transcripts of RBCS, GATA-2, GATA-11, and GATA-12 alongside increased GATA-9 expression. This convergence indicates a coordinated dark‑adaptation program, as captured by both methods.

The correlation analysis revealed strong internal consistency among the RNA‑seq‑derived metrics (DESeq2, FPKM, TPM), while agreement with the ΔΔCt method (RT‑qPCR) was weaker. This discrepancy likely reflects differences in sampling time points, reference gene stability, and methodological resolution. The highest correlation was observed between DESeq2 and ΔΔCt (Fig. 4). These findings demonstrate that although both platforms capture overlapping regulatory trends for certain GATA genes, each also reveals condition‑ and time‑specific expression changes. This underscores the value of integrating both approaches.

## Discussion

**Advantages of the PipeSeq program**

PipeSeq was developed with a focus on minimal system administration requirements and optimized for local execution on Windows‑based systems. The program offers a user‑friendly graphical interface that lowers the entry barrier, integrates state‑of‑the‑art data analysis methods, and is suitable for small to medium‑sized datasets on standard personal computers. This makes PipeSeq a practical solution for individual researchers and small laboratories that do not have significant computing resources or system administration experience.

Currently, there are several tools and platforms for transcriptomic analysis, such as Galaxy (Afgan et al., 2018), Nextflow (Di Tommaso et al., 2017), Snakemake (Mölder et al., 2021), as well as specialized pipelines like HISAT2-StringTie-Ballgown (Pertea et al., 2016) and Kallisto-Sleuth (Bray et al., 2016). Galaxy is a convenient web‑based platform with a graphical interface, but it requires separate server administration and is not always suitable for individual researchers or small laboratories without IT support (Afgan et al., 2018).

The Nextflow and Snakemake software platforms offer high flexibility and scalability through parallel task execution and support for containerization, which ensures complete analysis reproducibility. However, using these systems requires programming and Linux environment administration skills, limiting their use by biologists without console and scripting experience (Di Tommaso et al., 2017; Mölder et al., 2021).

An important aspect is support for differential gene expression analysis. Modern approaches recommend using packages based on the negative binomial distribution (e. g., DESeq2), which provide high accuracy and false discovery rate control (Zhao S. et al., 2020; Zhao Y. et al., 2021; Elahimanesh, Najafi, 2024).

Our developed PipeSeq software pipeline (Fig. 1) uses one of the fastest tools for read alignment (HISAT2) and transcript assembly (StringTie), which significantly outperform previous methods (TopHat and Cufflinks) in terms of speed and computational resource requirements (Kim et al., 2015, 2019). The integration of DESeq2 allows to automatically obtain statistically valid results of differential expression with FDR control (Love et al., 2014). This places it on par with recognized solutions in RNA‑seq data analysis, such as the HISAT2‑StringTie‑Ballgown pipeline, which uses a similar statistical approach, albeit less integrated (Pertea et al., 2016). Another advantage of the developed program is the ability to process and visualize qPCR data using the ΔΔCt method and to conduct comparative statistical analysis (calculating the Pearson, Spearman, and Kendall correlation coefficients) with three RNA‑seq normalization methods – FPKM, TPM, and DESeq2.

The developed PipeSeq program has the following features. 1. User interaction with the program occurs exclusively through a graphical interface (five windows). 2. All data processing stages are integrated within a single program (no need to connect additional packages) and do not require an internet connection. 3. The program includes automated selection of trimming parameters (adapter removal, quality threshold, minimum read length) based on quality control at the input and output. 4. A built‑in module for processing qPCR data using the ΔΔCt method (import of threshold cycle (Ct) values, calculation of normalized expression, comparative statistical analysis based on RNA‑seq data processing results and qPCR results).

**Expression profiles of GATA genes in C. reinhardtii**

Light is one of the key regulators of gene expression in photosynthetic organisms. Through a comprehensive study, we have obtained the most complete expression profiles of GATA genes in C. reinhardtii, supplementing publicly available RNA‑seq data with our own experiments using the RT‑qPCR method. Despite the fact that currently the results of transcriptomic studies in most cases do not require additional verification (Coenye, 2021), we encountered a limited amount of relevant data (three datasets) in open databases, accompanied by brief descriptions of the growth conditions for the cultures selected for analysis.

The resulting RNA‑seq data (Fig. 2) for some GATA genes showed low statistical significance of changes (FDR > 0.05). Processing of the selected RNA-seq datasets using the Pipe‑ Seq program allowed us: (1) to identify and confirm a pair of constitutively expressed reference genes RPL19–RPL32 (Liu et al., 2012); (2) to determine expression profiles of the target genes in response to specific stimuli, with the aim of subsequently applying the RT‑qPCR method for precise quantitative assessment of these changes across an expanded range of experimental conditions.

Our findings (Fig. 3) confirm that changes in light conditions are a significant factor modulating GATA gene expression in photosynthetic organisms (Manfield et al., 2007; Naito et al., 2007; Luo et al., 2010; Schröder et al., 2023). A previous analysis of the protein interaction network (Virolainen, Chekunova, 2024) identified three functional clusters in which the 12 GATA factors of C. reinhardtii are likely involved.

The first functional cluster, consisting of the GATA-1, GATA-2, and GATA-10 genes, links photoreception (genes CHLAMYDOMONAS PHOTOLYASE HOMOLOG 1 (CPH1), SHOC2/SUR8-like LRR (CSL)), circadian regulation (genes of the RHYTHM OF CHLOROPLAST (ROC) family) and phosphorus metabolism (the PHOSPHATE STARVATION RESPONSE 1 (PSR1) gene), ensuring adaptation of the cell to light regimes.

The second cluster (GATA-9) is associated with the functioning of the aryl hydrocarbon receptor complex, which is activated under high light conditions, supporting detoxification processes in the cell.

The third cluster (GATA-3, GATA-4, GATA-5, GATA-6, GATA-7, GATA-8, GATA-10, GATA-11, GATA-12) coordinates nitrogen assimilation (via the NITRATE REDUCTASE (NIT1) gene), chromatin remodeling (via genes encoding histone deacetylases), DNA replication (via the gene encoding helicase (CrRuvBL1)), membrane transport and cell division (via genes encoding the Rab family protein (RABF1), kinesin‑like protein, and a subunit 4 of the cyclosome), showing pronounced light dependence.

The observed dynamics of GATA gene expression represent a coordinated response that integrates key metabolic processes during the cell cycle (Voigt, Münzner, 1987; Müller et al., 2017; Salomé, Merchant, 2019). The response of all studied GATA genes to light stimuli suggests the presence of a cross‑regulation mechanism among the three functional networks through by yet unidentified or uncharacterized genes and proteins.

The results of our studies allow us to make the first significant assumptions regarding the functions of GATA TFs in C. reinhardtii and green algae in general (Schwechheimer et al., 2022), laying the necessary foundation for future research.

A comparative analysis of the use of three RNA‑seq normalization methods (FPKM, TPM, DESeq2), and the ΔΔCt method based on RT‑qPCR data demonstrated the highest correlation in the DESeq2–ΔΔCt pair (Fig. 4). This finding confirms the literature reports on the high accuracy of tools based on the negative binomial distribution (Zhao S. et al., 2020; Zhao Y. et al., 2021; Elahimanesh, Najafi, 2024) and demonstrates the reliability of the ΔΔCt normalization method for RT‑qPCR data for accurate quantification of the expression levels of target genes (Livak, Schmittgen, 2001; Schmittgen, Livak, 2008; Shi, He, 2014; He et al., 2015; Coenye, 2021; Schröder et al., 2023). Collectively, both approaches reveal overlapping trends for a small number of GATA genes and condition‑, method‑, and time‑dependent differences. This underscores the value of integrating massive (RNA‑seq) and targeted (RT‑qPCR) approaches to obtain a more comprehensive understanding of gene expression dynamics.

## Conclusion

During the study, we developed and applied the PipeSeq pipeline. It includes automated steps for data loading, read alignment, and statistical processing of RNA‑seq data. Additionally, the program enables the analysis and visualization of data generated by both RNA-seq and RT‑qPCR methods. The results of our study showed that RNA sequencing and RT‑qPCR methods can reveal similar patterns of gene expression changes, but show differences in the effect size estimation and sensitivity in detecting expression changes.

The data obtained allow us to conclude that the GATA TFs in C. reinhardtii form three functionally specialized groups (clusters), the coordinated regulation of which constitutes a key mechanism ensuring proper progression of the cell cycle under changing environmental conditions. The expression profiles of the GATA-2, GATA-4, GATA-5, GATA-6, GATA-8, GATA- 10, GATA-11, and GATA-12 genes suggest their involvement in the regulation of light-dependent metabolic processes. The GATA-1, GATA-3, GATA-7, and GATA-9 genes are involved in switching metabolism during the light/dark and dark/light transitions. Future research on GATA TFs in C. reinhardtii should be aimed at search and verification of the target genes and interactions in regulatory networks, as well as confirmation of the predicted functions in response to changes in other cultivation conditions

The PipeSeq program has demonstrated its effective- ness in a comprehensive study of differential gene expression as a tool for conducting a full cycle of bioinformatic ana- lysis of RNA‑seq data with the ability to process RT‑qPCR data and perform comparative statistical analysis of the results obtained using different methods. The developed pipeline can be used to study the gene expression profiles of any research object.

## Conflict of interest

The authors declare no conflict of interest.
